# Supplementary material for: Neuropeptide B mediates female sexual receptivity in medaka fish, acting in a female-specific but reversible manner
Source: eLife. 2019 Aug 6;8:e39495. doi: 10.7554/eLife.39495 (PMC6684226; doi:10.7554/eLife.39495)
Supplement: Supplementary file 1. [file elife-39495-supp1.docx]

Supplementary File 1. Abbreviations of brain and spinal cord regions and brain nuclei.

| Abbreviation | Full name | Location |
| --- | --- | --- |
| Brain and spinal cord regions | |  |
| aDH | Anterior part of the dorsal horn |  |
| adSC | Anterodorsal region of the spinal cord |  |
| aVH | Anterior part of the ventral horn |  |
| Cb | Cerebellum |  |
| DH | Dorsal horn |  |
| Die | Diencephalon |  |
| dlMO | Dorsolateral region of the medulla oblongata |  |
| dmMO | Dorsomedial region of the medulla oblongata |  |
| Hyp | Hypothalamus |  |
| MO | Medulla oblongata |  |
| mpSC | Middle to posterior part of the spinal cord |  |
| MT | Midbrain tegmentum |  |
| mVH | Middle part of the ventral horn |  |
| OB | Olfactory bulb |  |
| OpN | Optic nerve |  |
| OT | Optic tectum |  |
| pDH | Posterior part of the dorsal horn |  |
| Pit | Pituitary |  |
| pVH | Posterior part of the ventral horn |  |
| SC | Spinal cord |  |
| Tel | Telencephalon |  |
| Th | Thalamus |  |
| VH | Ventral horn |  |
| vlMO | Ventrolateral region of the medulla oblongata |  |
| vmMO | Ventromedial region of the medulla oblongata |  |
| Brain nucleus | |  |
| CP | Central posterior nucleus | Thalamus |
| Dl | Lateral nucleus of the dorsal telencephalic area | Dorsal telencephalon |
| Dm | Medial nucleus of the dorsal telencephalic area | Dorsal telencephalon |
| DP | Dorsal posterior nucleus | Thalamus |
| DT | Dorsal tegmental nucleus | Midbrain tegmentum |
| gc | Central gray | Brain stem |
| IQ | Inferior oblique of the oculomotor nerve nucleus | Midbrain tegmentum |
| IR | Inferior rectus of the oculomotor nerve nucleus | Midbrain tegmentum |
| is | Isthmus nucleus | Midbrain tegmentum |
| lHd | Left dorsal habenula | Habenula |
| MR | Medial rectus of the oculomotor nerve nucleus | Midbrain tegmentum |
| MRN | Medullary reticular nucleus | Medulla oblongata |
| NAT | Anterior tuberal nucleus | Hypothalamus |
| NDLI | Diffuse nucleus of inferior lobe | Hypothalamus |
| NPT | Posterior tuberal nucleus | Hypothalamus |
| NRL | Lateral recess nucleus | Hypothalamus |
| NRP | Posterior recess nucleus | Hypothalamus |
| NVT | Ventral tuberal nucleus | Hypothalamus |
| OT | Optic tectum excluding the periventricular gray zone (layer 3) | Optic tectum |
| Pbl | Basal lateral preoptic nucleus | Preoptic area |
| PGc | Central part of the preglomerular nucleus | Hypothalamus |
| PGZ3 | Periventricular gray zone (layer 3) | Optic tectum |
| PMg | Gigantocellular portion of the magnocellular preoptic nucleus | Preoptic area |
| PMm | Magnocellular portion of the magnocellular preoptic nucleus | Preoptic area |
| PMp | Parvocellular portion of the magnocellular preoptic nucleus | Preoptic area |
| PPa | Anterior parvocellular preoptic nucleus | Preoptic area |
| PPp | Posterior parvocellular preoptic nucleus | Preoptic area |
| PPv | Ventral periventricular pretectal nucleus | Pretectum |
| ra | Raphe nucleus | Brain stem |
| rHd | Right dorsal habenula | Habenula |
| RS | Superior reticular nucleus | Brain stem |
| RT | Rostral tegmental nucleus | Midbrain tegmentum |
| TS | Semicircular torus | Midbrain tegmentum |
| ttb | Tectobulbar tract | Brain stem |
| VL | Ventrolateral nucleus | Thalamus |
| Vl | Lateral nucleus of the ventral telencephalic area | Ventral telencephalon |
| VM | Ventromedial nucleus | Thalamus |
| Vp | Posterior nucleus of the ventral telencephalic area | Ventral telencephalon |
| Vs | Supracommissural nucleus of the ventral telencephalic area | Ventral telencephalon |
| Vv | Ventral nucleus of the ventral telencephalic area | Ventral telencephalon |
