# Supplementary material for: Neuropeptide B mediates female sexual receptivity in medaka fish, acting in a female-specific but reversible manner
Source: eLife. 2019 Aug 6;8:e39495. doi: 10.7554/eLife.39495 (PMC6684226; doi:10.7554/eLife.39495)
Supplement: Supplementary file 2. [file elife-39495-supp2.docx]

Supplementary File 2. Primers used in this study.

| Target | Direction | Purpose | Sequence (5′ to 3′) |
| --- | --- | --- | --- |
| *npba* | Forward | Real-time PCR | ATCTGATTTCTCACAACTCCAT |
| *npba* | Reverse | Real-time PCR | GATCCCTTGATTTCTTGGGACA |
| *npba* | Forward | Genotyping of ΔERE mutants (gDNA PCR) | GGAAAGGCAATTCCATAAATGTGGA |
| *npba* | Reverse | Genotyping of ΔERE mutants (gDNA PCR) | GGGTTATGCTGCCCACACTC |
| *npba* | Forward | Genotyping of ΔERE mutants (CS) | CATAAATGTGGATTAACACGGAGT |
| *npba* | Reverse | Genotyping of ΔERE mutants (CS) | GCCCACACTCTGTGATACCTGA |
| *npba* | Forward | Genotyping of knockouts (gDNA PCR) | CAGCCGTTTCCACTGAGCCTCT |
| *npba* | Reverse | Genotyping of knockouts (gDNA PCR) | CAACACGCTGTTGATCTCCGTCT |
| *npba* | Forward | Genotyping of knockouts (CS) | TCCACTGAGCCTCTAAAGCGCA |
| *npba* | Reverse | Genotyping of knockouts (CS) | TCCGTCTCGCCGCTGTCTGA |
| *actb* | Forward | Real-time PCR | CCCCACCCAAAGTTTAG |
| *actb* | Reverse | Real-time PCR | CAACGATGGAGGGAAAGACA |
| *npbb* | Forward | Real-time PCR | AGTAAAGAGGCGGCCGAGA |
| *npbb* | Reverse | Real-time PCR | GCAGATGGCCATGTCTTTGA |
| *npbb* | Forward | Genotyping of knockouts (gDNA PCR) | GTGTTCCTCCGCGTCAAAGGT |
| *npbb* | Reverse | Genotyping of knockouts (gDNA PCR) | CCTCTTTACTGTCCGCCAGGA |
| *npbb* | Forward | Genotyping of knockouts (CS) | GCGTCAAAGGTGCCAGCTCTA |
| *npbb* | Reverse | Genotyping of knockouts (CS) | CGCCAGGACCTCCTCGGACT |
| *npbwr2* | Forward | Genotyping of knockouts (gDNA PCR) | CAACACAGCAGTCATCTATGTCAT |
| *npbwr2* | Reverse | Genotyping of knockouts (gDNA PCR) | ACTACCAAGTAGCGATCAATGCT |
| *npbwr2* | Forward | Genotyping of knockouts (CS) | GTCATCTATGTCATTCTGAAAGC |
| *npbwr2* | Reverse | Genotyping of knockouts (CS) | AAGTAGCGATCAATGCTCATAAC |

gDNA PCR, PCR on genomic DNA; CS, cycle sequence.
