# Supplementary material for: Neuropeptide B mediates female sexual receptivity in medaka fish, acting in a female-specific but reversible manner
Source: eLife. 2019 Aug 6;8:e39495. doi: 10.7554/eLife.39495 (PMC6684226; doi:10.7554/eLife.39495)
Supplement: Supplementary file 3. [file elife-39495-supp3.docx]

Supplementary File 3. Species names and GenBank accession numbers of the protein sequences used in this study.

| Protein | Species | Accession number |
| --- | --- | --- |
| NPBWR1 | Human (*Homo sapiens*) | NP_005276 |
| NPBWR1 | Monkey (*Macaca mulatta*) | NP_001181156 |
| NPBWR1 | Bovine (*Bos taurus*) | NP_776499 |
| NPBWR1 | Mouse (*Mus musculus*) | NP_034472 |
| NPBWR1 | Rat (*Rattus norvegicus*) | NP_001014784 |
| Npbwr1 | Chicken (*Gallus gallus*) | NP_001314722 |
| NPBWR2 | Human (*Homo sapiens*) | NP_005277 |
| NPBWR2 | Bovine (*Bos taurus*) | NP_776500 |
| Npbwr2 | Chicken (*Gallus gallus*) | NP_001314723 |
| Npbwr2a | Zebrafish (*Danio rerio*) | NP_001124241 |
| Npbwr2b | Zebrafish (*Danio rerio*) | NP_001025429 |
| OPRD1 | Human (*Homo sapiens*) | NP_000902 |
| OPRM1 | Human (*Homo sapiens*) | NP_001272453 |
| NPB | Human (*Homo sapiens*) | NP_683694 |
| NPB | Bovine (*Bos taurus*) | NP_776369 |
| NPB | Mouse (*Mus musculus*) | NP_695020 |
| NPB | Rat (*Rattus norvegicus*) | NP_695205 |
| Npb | Chicken (*Gallus gallus*) | AND73819 |
| Npb | Zebrafish (*Danio rerio*) | NP_001120841 |
| Npba | Medaka (*Oryzias latipes*) | NP_001295908 |
| NPW | Human (*Homo sapiens*) | NP_001092926 |
| NPW | Porcine (*Sus scrofa*) | NP_998951 |
| NPW | Mouse (*Mus musculus*) | NP_001093134 |
| NPW | Rat (*Rattus norvegicus*) | NP_695206 |
| Npw | Chicken (*Gallus gallus*) | AND73820 |
